# Is the Result Worthy the Effort?

**Published:** 1901-05

**Authors:** 


### Editorial.

### IS THE RESULT WORTHY THE EFFORT?

THE readers of this journal are presented in this number with another budget of letters and communications from the Foreign Relations Committee of the National Association of Dental Faculties. This does not materially differ from that published in the April number, and is practically a continuation of the disagreeable subject of fraudulent diplomas. As a mere matter of news it may be interesting, but it is questionable whether it is good policy to spread these details before the profession.

The Foreign Relations Committee was originally appointed to secure information of the status of collegiate institutions abroad for the information of colleges in the United States. The committee quickly enlarged its sphere of labor and began unearthing the fraudulent diploma mills working under the very lax laws of the State of Illinois. This was not contemplated in the original appointment; in fact, the existence of such diploma factories was entirely unknown outside of the city of Chicago, and, probably, not extensively there. So secretly had the men who managed these concerns worked, that, so far as known, not a single diploma was ever disposed of in the United States. Their field of operation was entirely confined to foreign states The work of the chairman of the Foreign Relations Committee in breaking up this nefarious traffic was of such a valuable character that the Faculties Association fully endorsed it and voted the necessary means to continue the work. It was supposed that this had been practically accomplished, and, with several of the principals in jail, that nothing more would be heard of fraudulent diplomas on this side of the water. This, however, seems to have been an erroneous calculation, for if we are to judge by the activity of United States Consul Worman, the investigation has only just begun, or, rather, perhaps, it has been transferred to continental Europe.

This transfer would possibly be a cause of satisfaction were it not for the fact that it gives the haters of the United States—and they are legion abroad—an opportunity to display their animosity to our institutions, and that with the most malevolent spirit.

As the case has stood from the first, the Foreign Relations Committee has been fighting not for our protection, but for the people of foreign nations. The fact is well known that it would practically be impossible in this country to practise upon one of these diplomas, and the people are not interested in the subject further than they would be in having all criminals placed where they could do no further harm.

While it is eminently proper to help our neighbors, it is a question whether this should be carried to the extent of doing ourselves a positive injury. That this is being accomplished is made evident by a careful reading of the partial report furnished by Consul Worman upon another page. The whole tendency seems to the writer to be to cause a wrong conception, in the foreign mind, of our methods of education, not only in dentistry, but in all other departments of scholastic training. Those familiar with Europe need not be told that a deep prejudice exists against all things originating in the United States, or America, as they please to term it. They will imitate our products, duplicate our inventions, but judge with extreme bitterness the race that produced these results. It is, therefore, certain that publicity given to these things that have occurred upon our soil simply opens the way for abuse, misrepresentation, and a further increase in the prejudice with those unable to discriminate between truth and falsehood.

This country, it is well known, has been heretofore, if not now, the dumping-ground for the criminals of Europe, and the men engaged in this nefarious traffic are foreigners, but this fact is not considered abroad. The evidence of this is to be found in all grades of crime, and it is to be noted in the correspondence published. Consul Worman says, "One of the deans of these institutions (Chicago) was sentenced to the penitentiary in Breslau, escaped to America, and was there forced to undergo a prolonged treatment in a penitential 'water-cure.'" The libeller of this country in Europe, however, takes no note of this fact. For his purpose it is sufficient that the deed be accomplished on American soil.

The average European, especially in Germany, cannot understand either our principle of government, or our educational methods. The idea of an independent government, or State sovereignty, within a central power is entirely foreign to his conceptions, and to have a system of education without a supreme head, an educational minister at the seat of government, would be equivalent to no education at all. To these the incorporation of a college by a State is to place said college in the ranks with tradesmen who are obliged to procure a similar assent to open a store. We are judged, naturally, by the customs of each country, and the decision will invariably be against us. On the other hand, while we entertain great respect for the thoroughness of educational methods in continental Europe, the conditions are not the same here, and the system of training must always remain different, for the simple reason that our methods are the outgrowth of our needs and of our environments, and, while subject to change, can never, probably, partake of the same character, although reaching the same end, as those used in older civilizations.

It is, therefore, with some hesitation that space is given for this matter in this journal, as it will be certain to intensify the prejudice already existing in continental countries.

It must be apparent that as these fraudulent concerns are not injuring us, and are conducted by foreigners, that the Foreign Relations Committee is practically doing police work for the nationalities of Europe at the expense of the Faculties Association. It is a question whether this is wise, especially as it leads directly to the most foul slanders of everything regarded here as of value in education.

It would seem almost impossible to give exactly the truth abroad when this subject is up for discussion. This is made plain in the letter of A. E. Miller, of Chicago, taken from the *Zahnarztliche Rundschau*, Berlin. He says, "The writer, after graduating from a high school, attended the universities of Berlin and Leipzig, and was, as student and teacher, connected with colleges here [supposed Chicago], and may, therefore, be considered a competent judge.

"The so-called dental colleges, dental schools, etc., are, as a rule, private enterprises, and, according to the will of the Legislature, are not founded in the interest of science and humanity without yielding pecuniary profit to the managers. The words, 'incorporated under the laws of the State,' do not mean that the institutions are State institutions, but that they have been founded like insurance companies, large hardware concerns, liquor enterprises, etc.

"Therefore, the doctor's degrees of the college here are not to be confounded with the state diplomas in Germany, but would rank with the apprentice certificates of any of the above-mentioned business concerns. . . . There is in every State 'a State Board of Dental Examiners,' whose duty it is, in the interest of public health, to see that no unqualified persons are permitted to practise dentistry."

Could there be under the guise of truth a more satisfactory method of inculcating error than this writer manages to invent for the purpose, apparently, of libelling his supposed country and its institutions. He makes it appear that dental colleges are alone incorporated by the State, when he knew, if he knew anything, that all educational and professional institutions must take the same course, and from that source receive authority to grant degrees.

"These dental schools are not founded in the interest of humanity, but are business projects and for profit." This will be news to the managers of dental colleges, with very rare exceptions. The majority are kept alive, if not with a dead loss, with only sufficient income to meet current outlay. This veracious writer, in dealing with the truth, should have stated that possibly the State of Illinois was unique in having an incorporation law differing, in all essential provisions, from other States, and as it stands, or did stand, is an ever open door to a repetition of all sorts of frauds, professional and otherwise.

The effect of such a missive published in a prominent German journal may readily be conceived. Probably not a reader but at once made up his mind that professional dental education in America was a roaring farce. Who can estimate the injury this writer has done to the educational work in this country?

We are presented in this collection with another sample of a different character from the foregoing. The writer of this evidently meant his screed as a peculiarly fine sample of sarcastic writing, or an exhibit of German humor; in any event, it resolves itself into a quite perfect specimen of Ananias culture in the make up of the author. It is taken from the *Basel Vorwärts,* March 12, 1901.

"The doctor factory in Philadelphia, which, as is well known, on the payment of several hundred dollars, helps every one to a doctor title, is now so crowded with work that, to furnish the paper for doctor diplomas, eight large paper-factories are working day and night uninterruptedly, and even then thousands of aspirants for the doctor title must be turned away daily or put off for future years. Among the applicants for the coveted title are numerous ladies; the modern American lady knows no higher ambition (or has no higher pride) than to place Dr. before her name."

Attempts to answer such a monumental prevaricator and wholesale libeller of American women would be out of place, but for those who know foreign thought and foreign lack of information concerning America it need not be stated that every word of this infamous screed would be accepted as truth by the Basel subscribers of that sheet. The truth is that not a single fraudulent diploma has been issued in Philadelphia since the notorious Buchanan, many years back, was jailed by the authorities. Not only this, but, outside of Chicago, the writer does not know of any diploma mills, and even there the proprietors are fast being housed in the penitentiary.

In view of this, why will the Foreign Relations Committee insist upon allowing these foreign libellers access to all the dental journals of this country. The INTERNATIONAL DENTAL JOURNAL reluctantly opened its pages, and would have declined to be a party to this had it not seemed necessary that the dental profession should be informed of the trend of affairs in this direction and the dangerous quagmire into which we are certainly drifting.

It is time, in the writer's judgment, for the Foreign Relations Committee to seriously consider whether it is not stepping beyond, and very far beyond, the duties contemplated by the Association of Faculties. The police authorities should treat these criminals as they do others and as the United States detective service did the other day in running down a foreign counterfeiter, who was found well equipped for manufacturing foreign bank-notes. These diplomas have been made here by foreign criminals for foreign consumption entirely, and, while we should give neighborly help, it is the duty of the foreign states to seek out and punish their own criminals, and not make them a charge upon innocent peoples, and it is certainly not the business of the dentists of this country to do this work for them.

The United States has its own civilization, its own methods of education, its own laws regulating this, and its own people to care for and educate, and it will brook no interference or insolence from the narrow-minded European whose view is limited to what his grandfather taught or his great-grandfather invented.

